# A GIS-based multi-criteria framework for mapping potential irrigated agricultural zones in newly reclaimed arid agroecosystem

**DOI:** 10.1371/journal.pone.0351546

**Published:** 2026-06-26

**Authors:** Ahmed S. Abuzaid, Hassan H. Abbas, Yousif K. El Ghonamy, Mostafa A. Mostafa, Nazih Y. Rebouh, Mohamed S. Shokr

**Affiliations:** 1 Soils and Water Department, Faculty of Agriculture, Benha University, Benha, Egypt; 2 Soils, Water, and Environment Research Institute (SWERI), Agricultural Research Center (ARC), Giza, Egypt; 3 Department of Environmental Management, Institute of Environmental Engineering, RUDN University, Moscow, Russia; 4 Soil and Water Department, Faculty of Agriculture, Tanta University, Tanta, Egypt; Indian Institute of Technology Bombay, INDIA

## Abstract

Geographic assessment of natural resources is a pillar for sustainable agriculture in newly developed agroecosystems. The current work provides a new framework to discriminate agricultural potential zones by integrating the analytical hierarchy process (AHP) with fuzzy logic under the geographic information system (GIS) platform. The study was conducted on 303.54 km^2^ (30354 ha) in the western Nile Delta fringes, Egypt. Topographic maps, field surveys, and laboratory analyses were employed to specify parameters characterizing terrain, soil, and groundwater qualities. The main criteria and their respective sub-criteria were ranked and weighted using the AHP. The GIS tools were employed to generate raster layers using ordinary kriging geostatistical models, normalize the thematic layers using fuzzy membership functions, and integrate the fuzzified layers with their AHP-derived weights using the weighted sum algorithm. Results revealed that the consistency ratio of all the developed pairwise comparison models did not exceed 10%, indicating the efficacy of AHP in allocating the specific contribution of each criterion. Salinity, sodicity, and depth were key parameters controlling soil performance; meanwhile, potential salinity and infiltration problems primarily determined the feasibility of groundwater irrigation. Among four major criteria, the greatest impact was due to groundwater quality (50%), followed by chemical soil quality (24%) and physical soil quality (21%), while slope had the least contribution (5%). The potentiality analysis indicated that the studied soils are promising since good-quality soils occupied more than 60% of the studied area. Groundwaters with good, marginal, and poor quality occupied 40, 23, and 37% of the total area, respectively. The overall potentiality map showed that 36, 26, and 38% of the studied area displayed high, moderate, and low potential for agricultural expansion, respectively. The integration of AHP with GIS tools (geostatistical analysis and fuzzy set) can enhance insight into sustainable land-use planning and suggest also timely cropping practices. Further investigations are advocated to quantify the suitable cropping patterns in the studied region.

## 1 Introduction

The ongoing increase in the global population poses a significant threat to the sustainability of food production, especially in developing countries [1,2]. Hence, in regions with limited rainfall, irrigation plays a pivotal role in addressing food insecurity and environmental instability [3]. Irrigation can increase crop yields twice compared with rainfed agriculture [4]. It can also provide a potent strategy to ameliorate micro-climate by reducing air and surface temperature and increasing humidity [3]. The irrigated farmlands cover 435 M ha (million hectares), accounting for 20% of the global agricultural lands [5]. However, these fields contribute to 40% of the world’s food production [6]. By 2050, cultivating an extra 200–1000 M ha would be necessary to meet the anticipated food demand [7]. Therefore, during the past century, large-scale irrigation projects have been launched in arid regions to convert marginal desert lands into croplands [8–10]. This step is imperative to achieve the United Nations Sustainable Development Goals (SDGs) like no poverty (SDG1), zero hunger (SDG 2), and life on land (SDG 15).

The arid lands (arid and semiarid) cover about 30% of the world’s land surface [11]. They are among the most fragile ecosystems, characterized by extreme climate conditions, poor soil quality, and limited water resources.[12]. Therefore, the cropland expansion into vast desert lands is challenging [13]. On regional scales, accurate delineation of potential agricultural zones is a pillar for sustainable development planning [14,15]. This process addresses diverse interfering factors related to topography, soil, and irrigation water quality [16,17]. In this context, multicriteria decision analysis (MCDA) serves as an efficient tool to decompose such a complex issue [18,19]. The analytical hierarchy process (AHP) provided by Saaty [20] is a potent MCDA technique to prioritize multicriteria based on expert opinions. The AHP explains the interactions among various parameters according to their relative influence [21,22]. Thus, adopting this valuable technology when assessing natural resources would help in securing agroecosystem functions.

In large-scale studies, depending solely on AHP is not adequate to assess natural resources [10,23] since various spatial and non-spatial data are analyzed, causing a big challenge for decision-making [18]. Thus, the GIS (geographic information system) has been efficiently employed in multicriteria analysis to manage and monitor natural resources [14,24,25]. The GIS is versatile technique utilized to capture, save, manipulate, and compile extensive spatial and non-spatial data [23,26], achieving accurate representations for natural resources [27]. The spatial analysis tools within GIS software are employed to create thematic layers and facilitate digital or predictive mapping [12,28]. The GIS-fuzzy sets can handle uncertainty due to diverse reference and ranking criteria besides the subjectivity arising from applying simple and supervised classifiers [29–31]. Thus, combining AHP with fuzzy logic under the GIS platform can preclude uncertainties in expert opinions, providing reliable evaluations [30]. In semi-arid croplands, such integration has led to dependable field crop suitability assessment in Iran [32,33] and Turkiye [29]. Moreover, it has facilitated the development of fast, cost-effective, and precise fertility maps for corn production in Faris Province, south Iran [34]. Yet, the feasibility of this potent framework under locally dominant conditions, especially in arid regions is still arguable.

Egypt, with a total land area of one million km^2^ (100 M ha), is one of the most arid countries on the planet. Based on official statistics [35], the total cropland area is 4.06 M ha depending entirely on irrigation. The Nile River serves as the main source of freshwater in Egypt and provides a fixed annual share of 55.5 billion cubic meters, making up 93% of freshwater and 68% of the total water resources in the country. The agricultural sector receives more than three-quarters of the water budget [35]. Therefore, depending on groundwater resources, mega land reclamation projects have been launched during the last three decades in the western desert, which accounts for two-thirds of the total land area of Egypt [36]. Besides improving national food security, these projects are anticipated to add positive socioeconomic benefits by alleviating the pressure on old fertile lands along the Nile River and providing new job opportunities [15]. Yet, developing new desert croplands using full groundwater irrigation is still arguable, advocating for reliable appraisal of land and water resources.

In Egypt, studies have applied GIS-fuzzy and AHP technologies to assess land resources in desert regions. For instance, Abuzaid, Mazrou [10] developed land quality indices in Matruh Governorate using a hybrid GIS-AHP approach. Yet, their focus was limited to land (soil and terrain) resources, with minimal consideration of groundwater potential. Given the increasing reliance on non-renewable groundwater aquifers (e.g., Nubian Sandstone, Moghra, and Fissured Carbonate) for irrigation in large-scale reclamation projects, there is a pressing need to evaluate both landscape and water resources comprehensively. This is because both the quantity and quality of groundwater is highly dynamic and depends mainly on withdrawal rates [15,37]. Thus, the present study proposes a novel framework that integrates AHP, fuzzy logic, and geostatistical analysis within a GIS environment to assess the agronomic potential of newly reclaimed desert lands in the western Nile Delta, Egypt. The framework aims to support sustainable agricultural development and inform future land-use planning in similar arid regions.

## 2 Materials and methods

### 2.1 Site description

**Fig 1:**
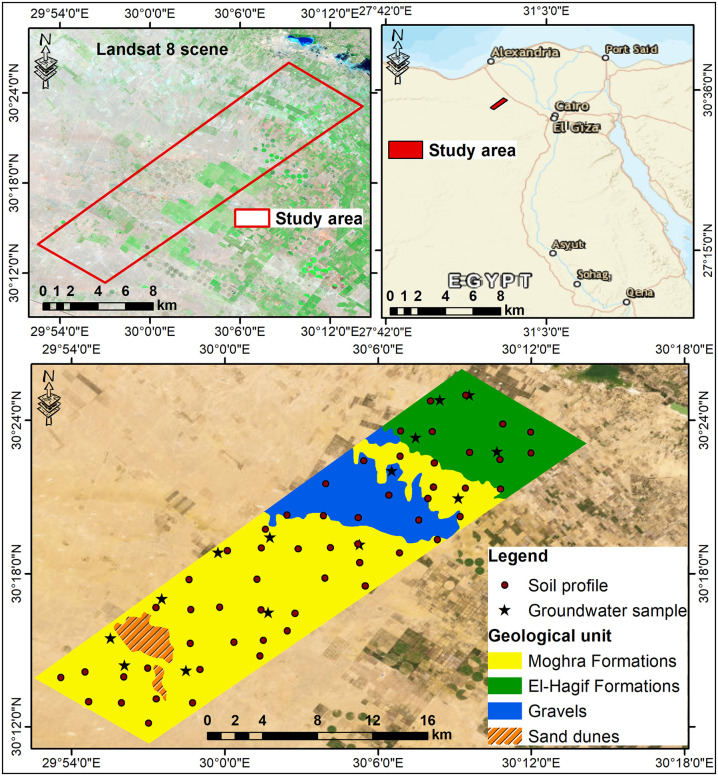
Location maps of the studied region.

The studied region covers 303.54 km^2^ (30354 ha) in the western Nile Delta fringes. The geographic location is in the UTM (Universal Transverse Mercator) zone 36 N between 30° 11′ 14ʺ to 30° 25′ 52ʺ N and 29° 52′ 30ʺ to 30° 14′ 08ʺ E (Fig 1). Materials from plants, animals, or other natural environments are not collected for the study. There was also no need for special licenses for field site access or research operations because the study was conducted on publically accessible property and did not include interactions with regulated species or ecosystems. The climate data collected from Wadi El-Natrun station revealed that January is the coldest month with a minimum temperature of 8 °C, while July is the hottest month with a maximum temperature of 36 °C. The mean annual temperature is 21 °C and the region receives a total annual rainfall of 43 mm. This implies that the soil temperature regime is “Thermic” and the soil moisture regime is “Torric” [38],

The elevation ranges from –14–174 m above sea level and the slope gradient varies from 0 to 16%. According to CONCO-Coral/EGPC [39], the sedimentary succession spans from the Miocene to the Quaternary eras. The lower Miocene Moghra Formations are composed of sand, silt, and clay mixed with minor carbonate interbeds and cover 63.86% of the total area. The Late Tertiary (Pliocene) sediments represented by El Hagif Formations are composed of limestone with marl inter-beds and occupy 19.64% of the total area. The Quaternary deposits cover 16.50%, including gravels (13.55%) and sand dunes (2.95%). The groundwater from the Moghra aquifer is the main source of irrigation.

### 2.2 Geomorphic mapping

One scene (path 177/row 39) of Landsat 8 was obtained through United states Geological Survey (USGS) Earth Explorer gateway in August 2022 (https://earthexplorer.usgs.gov/). The digital preprocessing and processing of satellite image was accomplished using the ENVI 5.1 software [40]. The preprocessing included atmospheric correction using the Fast Line-of-sight Atmospheric Analysis of Spectral Hypercubes (FLAASH) module, stretching, band stacking, and spatial and spectral subsets. The image processing involved unsupervised classification through ISO DATA classifier model followed by supervised classification through maximum likelihood model. Four topographic map sheets (1: 50000) covering the studied region were scanned. Using ArcGIS 10.8 software (ESRI Co, Redlands, USA) the maps were geo-referenced to the Universal Transverse Mercator (UTM) projection, Zone 36 North, and WGS- 84 datum. Thereafter, the spot heights as well as contour lines in every 10 m interval were digitized and subjected to topo to raster interpolation technique to create a raster DEM (digital elevation model) with a spatial resolution of 10 m. The processed satellite imagery, DEM, and geological map were overlain to extract the geomorphic units as suggested by Zinck, Metternicht [41].

### 2.3 Field work and laboratory analysis

Fifty-six geo-referenced soil profiles (Fig 1) were randomly distributed across the studied region to represent geological features. During August of 2022, the soil profiles were dug to a 150-cm depth or a lithic contact. The morphological features of each profile were identified based on the Food and Agriculture Organization of the United Nations (FAO) [42] and Soil Science Division Staff [43]. A total of 163 disturbed soil samples were gathered from the subsequence horizons, kept in polyethylene bags, and transported to the laboratory. Undisturbed soil cores were also obtained to determine the soil bulk density (BD). In addition, fourteen groundwater samples were collected from available artesian wells. *In situ* measurements of water EC (electrical conductivity) and pH were accomplished using a portable HACH instrument (HQ 40d, multi, USA). Groundwater samples were then collected using 500 mL high-density polypropylene vials and transported to the laboratory. Further water analyses were done following standard methods [44].

The soil analyses were conducted following standard methods [45]. The samples were air-dried, crushed, and sieved using a 2-mm mesh and the volume of CF (coarse fragments; > 2 mm) was recorded. The determined physical characteristics were particle size distribution (pipette method), available soil water content (AWC), and hydraulic conductivity (HC). The chemical analyses involved pH (in 1:2.5 soil-water suspensions), EC (in soil paste extracts), OM (organic matter) content using the Walkley-Black procedure, ESP (exchangeable sodium parentage) using the ammonium acetate method, lime content using the calcimeter, and gypsum content using acetone precipitation method.

### 2.4 Modeling land and groundwater potentiality

**Fig 2:**
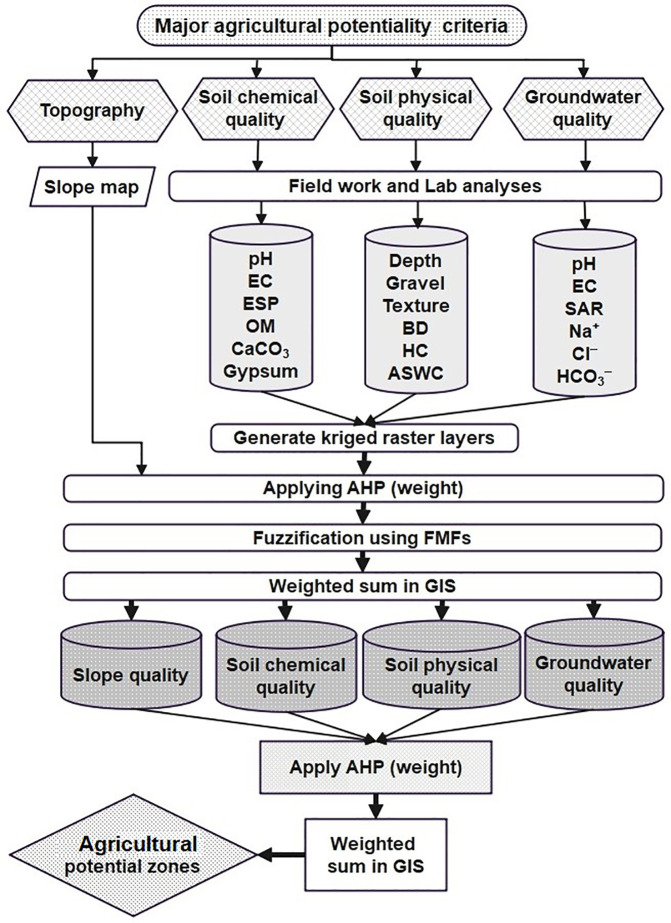
Schematic outline of the methodology applied for modeling agricultural potentiality. (EC, electrical conductivity; ESP, exchangeable sodium percentage; OM, organic matter; BD, bulk density; HC, hydraulic conductivity; ASWC, available soil water content; SAR, sodium adsorption ratio; FMF; fuzzy membership function).

This procedure included (1) generating raster layers, (2) thematic layer standardization, (3) weighting process, and (4) developing the final agricultural potentiality map (Fig 2).

#### 2.4.1 Generating raster layers.

The common criteria determining the ability of terrain, soil, and groundwater to sustain agroecosystem services were selected based on available literature [38,42,43,46–48]. Using ArcGIS 10.8 software, the geostatistical analysis was implemented to generate raster layers for soil and groundwater attributes using the ordinary kriging (OK) technique. The OK describes the spatial structure of environmental data according to the regional spatial scale, distance between locations, and spatial pattern of semivariograms. This technique can provide unbiased and optimal prediction since it based on various semivariograms to minimize the variance of prediction errors and display numerous map outputs [12,30]. The predicted value at unsampled location Z(*x*_0_) is estimated based on measured data (Z(*xi*)), weights of measured values (λ_*i*_) within a certain distance, and the number of predicted values (*n*) within certain neighbor samples according to Eq 1 as follows:


$$\begin{array}{c} Z_{\left(x\text{0}\right)}=\sum_{i=\text{1}}^{n}\lambda_{i}\times Z_{\left(xi\right)} \end{array}$$ (1)

The normality of data was checked using the normal Q-Q (quartile-quartile) plots and the histograms were also explored to define distribution outliers. The skewed data were transformed using logarithmic and Box–Cox methods to minimize the effects of outliers and address the non-normality. Thereafter, the theoretical semivariogram models (e.g., spherical, exponential, Gaussian … etc.) was fitted to the empirical models computed from the data. The best-fitted models with lower prediction errors were used for map production.

#### 2.4.2 Thematic layer standardization.

The kriged raster layers were normalized using FMFs (fuzzy membership functions) in ArcGIS 10.8 software. In this step, each pixel in the kriged layers was assigned a score value between 0 (the worst condition) to 1.0 (the best condition). As shown in Table 1, the applied functions included FMF1 (linear-increasing; Eq 2), FMF2 (linear-decreasing; Eq 3), and FMF3 (near). The FMF1 and FMF2 calculate memberships based on the user-inputted upper (U) and lower (L) limits for a variable (x) as follows:

**Table 1:** Criteria and scoring functions used for quantifying agricultural potentiality.

Main criterion	Sub-criterion	FMF	Lower limit	Upper limit	Midpoint	Spread	Reference
Topography	Slope, %	FMF2	2	30	---	---	[42]
Soil chemical quality	pH	FMF3	---	---	7	0.1	[43]
	EC, dS m^–1^	FMF2	2	16	---	---	
	ESP	FMF2	15	50	---	---	[47]
	OM, g kg^–1^	FMF1	0	25	---	---	[48]
	CaCO_3_, g kg^–1^	FMF2	20	250	---	---	[42]
	Gypsum, g kg^–1^	FMF2	0	60	---	---	
Soil physical quality	Depth, cm	FMF1	25	150	---	---	
	CF, %	FMF2	0	40	---	---	
	Sand, %	FMF2	0	70	---	---	[10]
	Silt, %	FMF2	0	50	---	---	
	Clay, %	FMF1	0	30	---	---	[49]
	BD, Mg m^–3^	FMF2	1.2	1.8	---	---	[48]
	ASWC, %	FMF1	10	20	---	---	
	HC, cm h^–1^	FMF3	---	---	6	0.1	
Groundwater quality	pH	FMF3	---	---	7	0.1	[46]
	EC, dS m^–1^	FMF2	0.7	3	---	---	
	SAR	FMF2	3	9	---	---	
	Na, mmol_C_ L^–1^	FMF2	5	20	---	---	
	Cl, mmol_C_ L^–1^	FMF2	5	20	---	---	

EC, electrical conductivity; ESP, exchangeable sodium percentage; OM, organic matter; CF, coarse fragments; BD, bulk density; ASWC, available soil water content; HC, hydraulic conductivity; SAR, sodium adsorption ratio; FMF, fuzzy membership function; FMF1, linear-increasing; FMF2, linear-decreasing; FMF3, near


$$\begin{array}{c} \text{FMF1}=\left\{ \begin{array}{c} @l\;\text{1}\;if\;x\;\geq U\\ \frac{x-L}{U-L}\;if\;L<x<U\\ \;\text{0}\;if\;x\;\leq L \end{array}\right.\; \end{array}$$ (2)

$$\begin{array}{c} \text{FMF2}=\left\{ \begin{array}{c} @l\;\text{1}\;if\;x\;\leq L\\ \frac{U-x}{U-L}\;if\;L<x<U\\ \;\text{0}\;if\;x\;\geq U \end{array}\right.\; \end{array}$$ (3)

The FMF3 computes memberships for values, which are close to an intermediate level. The score is 1 at the midpoint and declines to zero when being far from the midpoint. This function is implemented using user-inputted midpoint (f1) and spread (f2) values according to Eq 4 as follows:


$$\begin{array}{c} \text{FMF3}=\frac{\text{1}}{\left(\text{1}+{\text{f}}_{\text{1}}\right)\times {\left(\text{x}-f_{\text{2}}\right)}^{\text{2}}} \end{array}$$ (4)

#### 2.4.3 Weighting procedure.

As suggested by Saaty [50], the AHP procedure was implemented in order to rank and weight the main and sub-criteria affecting local cropland productivity. In the first step, a pairwise comparison matrix with three criteria (3 × 3), including slope, soil, and groundwater was designed. Using a rating scale from one to nine, the relative importance of each main factor over another was set based on the insight into ten local agricultural experts besides the authors’ experiences. In the same manner, additional comparison models (n × n) were designed to rank the sub-criteria quantifying soil and groundwater qualities. The AHP-OS (AHP online system package) was run to compute the weighting factor. To test the validity of the AHP results, the consistency ratio (CR) was considered, where matrices achieving CR ≤ 0.1 (10%) were accepted.

#### 2.4.4 Generating agricultural potentiality map.

To delineate the potential of soil and groundwater factors, the fuzzified raster layers of sub-criteria describing each factor were complied with their weights using the weighted sum algorithm under the GIS platform. Thereafter, the layers were reclassified into three classes (poor, marginal, good) using Jenks’s natural breaks classifier [51]. The Jenks method optimizes value arrangement into categories as it minimizes within-class variance but maximizes between-classes variance [30]. In the same manner, to generate the overall potentiality maps, the fuzzified slope layer was integrated with soil chemical quality, soil physical quality, and groundwater quality using the weighted sum model. Based on the final index value, the potentiality map was reclassified into three classes (low, moderate, high) using the Jenks classifier. The raster to polygon tool was utilized to convert raster datasets to polygon features, and finally the area of each potentiality class was calculated.

### 2.5 Model validation

The efficacy of the developed framework was tested via examining two levels of accuracy. The first one was linked to the reality of AHP results by testing the CR; meanwhile, the second level focused on assessing the performance of predictive mapping techniques. The AHP was done twice utilizing the arithmetic and geometric mean algorithms of the expert judgments, and the method yielding the lowest CR was considered for the next phase. The cross-validation technique in the light of prediction errors was employed to evaluate the OK models used for creating the thematic layers. The models achieving the prerequisites of dependable prediction were selected for digital mapping. The accepted models demonstrated the lowest value of mean error (ME) and mean standardized error (MSE), similar values of root mean square error (RMSE) and average standardized error (ASE), and root mean square standardized error (RMSSE) close to unity. The mathematical expressions of prediction errors are as follows:


$$\begin{array}{c} \text{ME}=\frac{\text{1}}{n}\sum_{i=\text{1}}^{n}\left(\text{Z}\left(y_{i}\right)-\text{Z}\left(x_{i}\right)\right) \end{array}$$ (5)

$$\begin{array}{c} \text{MSE}=\frac{\text{1}}{n}\sum_{i=\text{1}}^{n}\;\left|\text{Z}\left(y_{i}\right)-\text{Z}\left(x_{i}\right)\right| \end{array}$$ (6)

$$\begin{array}{c} \text{RMSE}=\sqrt{\frac{\text{1}}{n}\sum_{i=\text{1}}^{n}{\left(\text{Z}\left(y_{i}\right)-\text{Z}\left(x_{i}\right)\right)}^{\text{2}}} \end{array}$$ (7)

$$\begin{array}{c} \text{ASE}=\sqrt{\frac{\text{1}}{n}\sum_{i=\text{1}}^{n}{\left|\left(\text{Z}\left(y_{i}\right)-\sum_{i=\text{1}}^{n}\frac{\text{Z}\left(x_{i}\right)}{n}\right)\right|}^{\text{2}}} \end{array}$$ (8)

$$\begin{array}{c} \text{RMSSE}=\sqrt{\frac{\text{1}}{n}\sum_{i=\text{1}}^{n}{\left|\text{Z}\left(y_{i}\right)-\text{Z}\left(x_{i}\right)\right|}^{\text{2}}} \end{array}$$ (9)

where, Z(*x*_i_) and Z(*y*_i_) denote the measured and predicted value, respectively and n is the number of sampling locations.

## 3 Result

### 3.1 Geomorphology and soils

**Fig 3:**
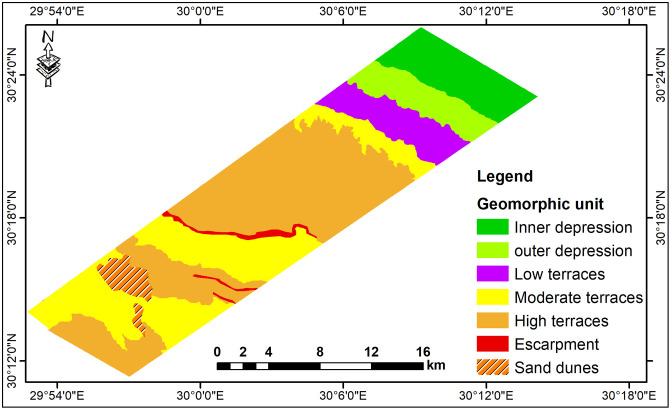
Geomorphic map of the studied area.

As shown in Table 2 and Fig 3, the studied area includes four main landforms: depression, terraces, sand dunes, and remnant hills. The low-lying lands (inner and outer parts of the depression) occurred in the northeastern parts, covering 17.98% of the total area. A series of terraces covered more than three-quarters of the studied area (77.56%), including low, moderate, and high terraces. These units represented 7.78, 27.11, and 42.67% of the total area, respectively. These terraces occupy the middle and southwestern parts of the studied region. Longitudinal sand dunes occupied 2.96% of the total area and occurred mainly in the southwestern parts. A steep escarpment of remnant hills occurred in the middle parts and covered 1.5% of the total area. These hills occurred in the middle parts of the studied region. The soils belonged to two orders: Entisols and Aridisols. The Entisols dominated the area, where 89.09% of the soils belonged to this order and were classified as Lithic Torripssaments (1.82%), Typic Torriorthents (41.82%), and Typic Torripssaments (45.45%). The Aridisols covered the remaining 10.91% and were classified as Lithic Haplocalcids (5.45%) and Typic Haplosalids (5.45%).

**Table 2:** Geomorphic units and soil taxonomy of the studied area.

Lithology	Landform	Unit	Area		Soil Taxonomy
			km^2^	%	
El-Hagif Fm.	Depression	Inner	32.22	10.61	Typic Haplosalids, 50%
					Typic Torripsamment, 50%
		outer	22.34	7.36	Typic Torriorthents, 75%
					Typic Torripsamment, 25%
Moghra Fm. and gravel	Series of terraces	Low	23.62	7.78	Typic Torripsamment, 75%
					Typic Torriorthents, 25%
		Moderate	82.30	27.11	Typic Torriorthents, 50%
					Typic Torripsamment, 37.5%
					Lithic Haplocalcids, 6.25
					Lithic Torripsamment, 6.25
		High	129.53	42.67	Typic Torripsamment, 50%
					Typic Torriorthents, 42.3%
					Lithic Haplocalcids, 7.7%
Quaternary deposits	Sand dunes	Longitudinal sand dunes	8.98	2.96	---
Moghra Fm.	Remnant hills	Escarpment	4.54	1.50	---

### 3.2 Soil and groundwater properties

Results in Table 3 reveal that the soils were very shallow to very deep with a depth varying from 15 to 150 cm. They had very few to many CF with a content ranging from 0.3 to 28.54% [42]. The soil pH varied from 7.33 to 8.80, indicating that the soils were neutral to strongly alkaline [43]. The EC ranged from 0.2 to 86.76 dS m^–1^, reflecting a wide salinity range from non-saline to strongly saline [43]. The ESP ranged from 2.57 to 54.30, indicating none to very high sodicity hazards [47]. The soils had extremely low organic matter content not exceeding 7 g kg^–1^ [48]. The lime contents ranged from 0.30 to 841.50 g kg^–1^, indicating that the soils were slightly to extremely calcareous [42]. The gypsum content ranged from 0.20 to 59.70 g kg^–1^, indicating that the soils were none to slightly gypsiric [42]. The particle size distribution was dominated by sand with an average of 77.18% followed by silt (18.41%) and clay (4.41%). The BD was moderate to very high with a range of 1.52 to 1.93 Mg m^–3^. The AWC was low to medium with a range of 6.40 to 17.30%. The soil had a low to very high HC with a range of 1.89 to 16.92 cm h^–1^.

**Table 3:** Descriptive statistics of soil properties.

Property	Unit	Min	Max	Mean	SD	CV, %
Depth	cm	15.00	160.00	137.85	26.28	19.06
Gravel	%	0.03	28.54	2.32	3.32	143.07
pH	---	7.33	8.80	8.04	0.31	3.82
EC	dS m^–1^	0.20	86.76	5.37	9.25	172.32
ESP	---	2.57	54.30	9.50	7.24	76.20
OM	g kg^–1^	0.00	6.50	2.38	1.38	57.84
CaCO_3_	g kg^–1^	0.30	841.50	49.07	88.39	180.14
Gypsum	g kg^–1^	0.20	59.70	20.08	11.97	59.60
Sand	%	38.70	96.90	77.18	13.50	17.50
Silt		1.50	59.70	18.41	13.65	74.15
Clay		0.50	27.30	4.41	3.30	74.76
BD	Mg m^–3^	1.52	1.93	1.72	0.09	5.14
ASWC	%	6.40	17.30	9.70	2.74	28.27
HC	cm h^–1^	1.89	16.29	7.00	3.28	46.78

See footnote of Table 1, SD, standard deviation; CV, coefficient of variation.

The descriptive statistics of chemical composition of groundwater samples is shown in Table 4. The FAO 29 guidelines for interpreting water quality for irrigation are shown in the supplementary data (S1 Table in S1 File) pH range was slightly higher than the permissible spectrum for safe irrigation (6.5–8.4) as suggested by FAO 29 guidelines [46]. The EC ranged from 0.59 to 3.98 dS^–1^, indicating none to severe potential salinity hazards. The infiltration problem is assessed through considering values of EC and SAR since insufficient Ca^2+^ in water exacerbates soil dispersion by Na^+^ [46]. When assessing potential infiltration problems using EC and SAR together, the groundwater displayed none to moderate restrictions. In terms of toxicity hazards due to the root intake, the SAR ranged from 2.51 to 7.06, demonstrating none to moderate problems. Moreover, the concentrations of Cl^–^ varied from 3.73 to 34.40 mmol_C_ L^–1^, indicating none to severe toxicity problems. On the other hand, the concentrations of both Na^+^ and Cl^–^ in all water samples exceeded the permissible limits of 3 mmol_C_ L^–1^ for leaf absorption by sensitive crops. Conversely, the concentrations of HCO_3_^–^ stood below the safe limits of 1.5 mmol_C_ L^–1^ for overhead sprinkler irrigation.

**Table 4:** Descriptive statistics of groundwater properties.

Property	Unit	Min	Max	Mean	SD	CV, %
pH	---	7.12	8.85	7.98	0.39	4.83
EC	dS m^–1^	0.59	3.89	1.18	0.87	73.79
Ca^2+^	mmol_C_ L^–1^	1.52	12.60	3.60	2.89	80.38
Mg^2+^		0.42	5.05	1.81	1.33	73.43
Na^+^		3.10	21.00	6.21	4.60	74.13
K^+^		0.11	0.31	0.20	0.05	25.86
Cl^–^		3.73	34.40	9.02	7.94	88.04
SO_4_^2–^		1.00	4.58	2.05	0.96	47.05
HCO_3_^–^		0.21	1.08	0.74	0.31	42.09
SAR	---	2.51	7.06	3.66	1.10	30.10

See footnote of Table 1.

### 3.3 Agricultural potentiality evaluation

**Fig 4:**
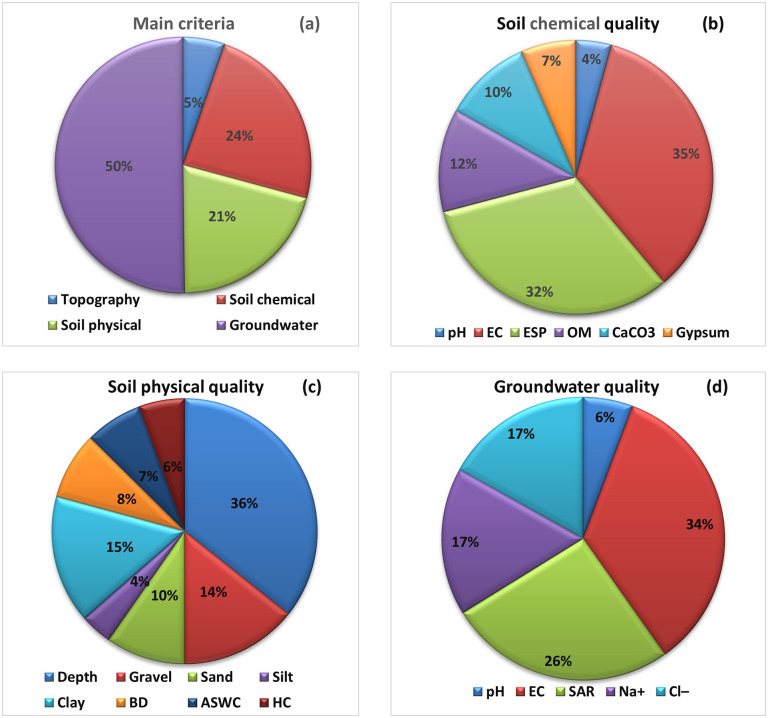
Contributions of main (a) and sub-criteria (b, c, and d) to cropland potential. (EC, electrical conductivity; ESP, exchangeable sodium percentage; OM, organic matter; BD, bulk density; ASWC, available soil water content; HC, hydraulic conductivity).

**Fig 5:**
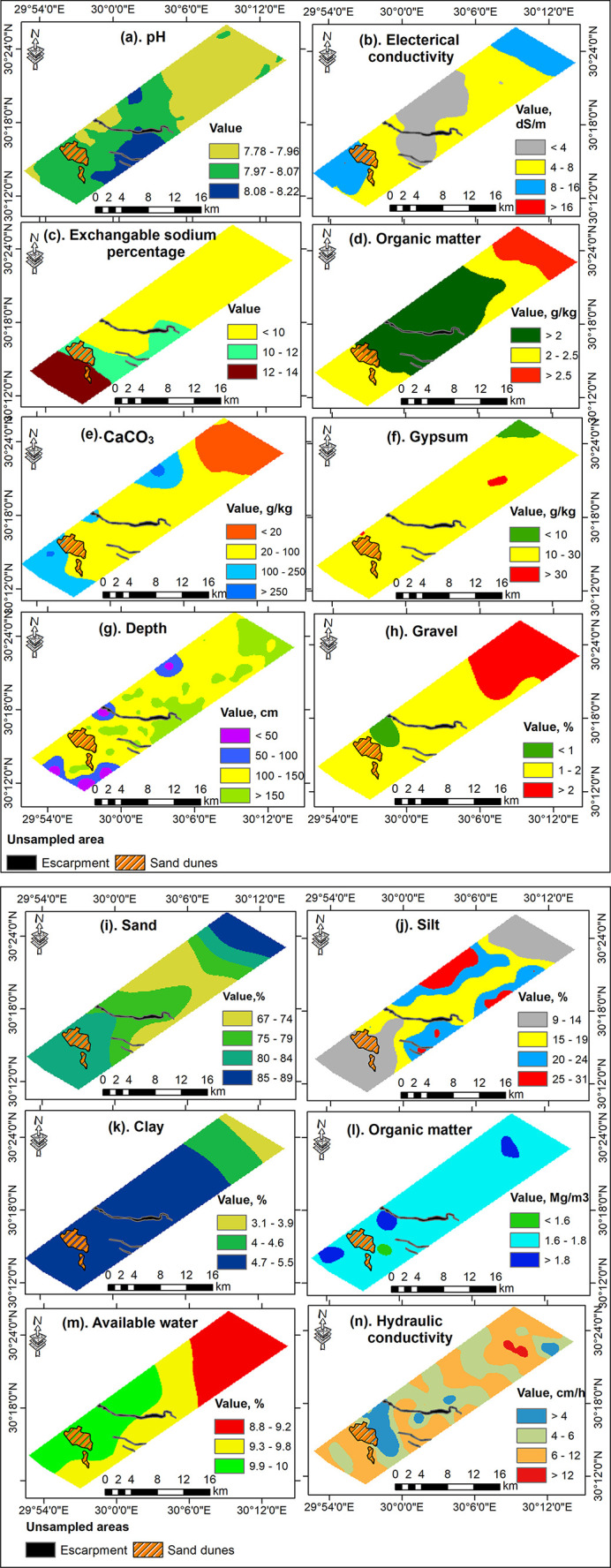
Spatial distribution maps of soil properties.

**Fig 6:**
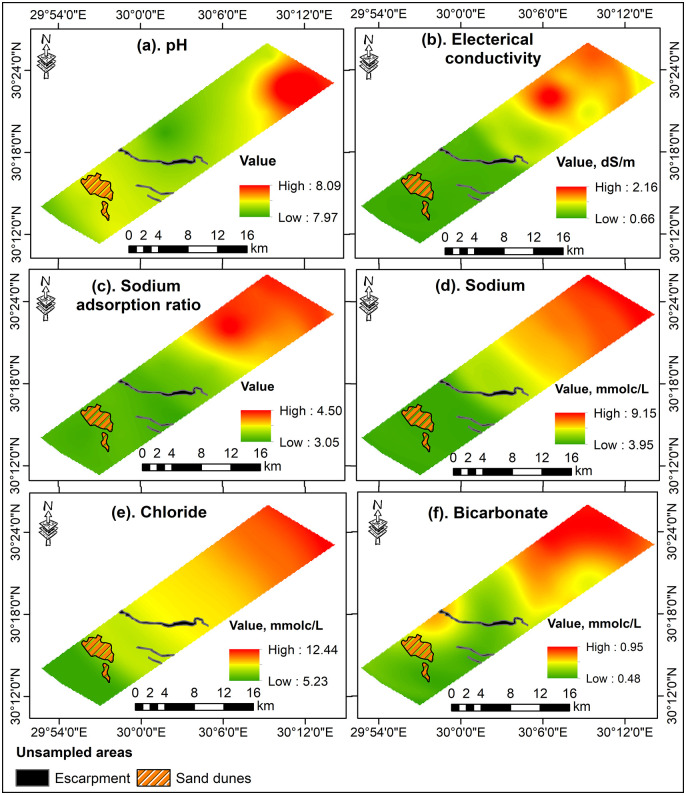
Spatial distribution maps of groundwater attributes.

The AHP for major and sub-criteria adopted to assess agricultural potentiality in the studied area are presented in Table 5 and the weight values are given in Fig 4. The EC and ESP were key factors governing soil chemical quality with a total effect of 67%, while pH had the lowest impact (only 4%). The spatial distribution of soil properties is explored through semivariograms (S1 Fig in S1 File). The cross-validation of the selected semivariogram models (S2 Fig in S1 File) reveals good association between the estimated and measured values. The kriged maps, shown in Fig 5, illustrate that EC and lime content showed higher spatial variations over the studied area compared with other properties. Yet, the slightly saline (EC 4–8 dS m^–1^) and moderately calcareous (20–100 g CaCO_3_ kg^–1^) soils were predominant in the middle parts. The soil depth and silt content had the highest and lowest contributions to soil physical quality with a relative importance of 36 and 4%, respectively. Maps reveal that deep (100–150 cm) and very deep (> 150 cm) soils dominated the studied area. The shallow (100–50 cm) and very shallow (< 50 cm) soils occurred mainly in areas with high lime content. For irrigation purposes, both EC and SAR contributed to 61% of groundwater suitability. The spatial distribution of groundwater quality attributes is explored through semivariograms (S3 Fig in S1 File). The cross-validation of the selected semivariogram models (S4 Fig in S1 File) reveals good association between the estimated and measured values. As shown in Fig 6, the intensity of potential salinity, infiltration, and toxicity hazards tended to increase northeastwards.

**Table 5:** Comparison matrixes and weights of criteria used for agricultural potentiality evaluation.

Criterion	Pairwise comparison matrix								Rank	Weight
	(1)	(2)	(3)	(4)	(5)	(6)	(7)	(8)		
Main factor										
(1) Topography	1	1/5	1/4	1/8					4	0.053
(2) Soil chemical quality	5	1	1	1/2					2	0.239
(3) Soil physical quality	4	1	1	1/3					3	0.206
(4) Groundwater quality	8	2	3	1					1	0.502
Consistency ratio (CR) = 0.001									Sum	1.000
Soil chemical quality										
pH	1	1/8	1/4	1/4	1/3	1/2			6	0.042
EC	8	1	1	4	4	4			1	0.347
ESP	4	1	1	4	4	4			2	0.32
OM	4	1/4	1/4	1	1	3			3	0.122
CaCO_3_	3	1/4	1/4	1	1	2			4	0.104
Gypsum	2	1/4	1/4	1/3	1/2	1			5	0.065
Consistency ratio (CR) = 0.039									Sum	1.000
Soil physical quality										
Depth	1	4	4	5	4	4	4	6	1	0.357
Gravel	1/4	1	2	2	2	2	2	2	3	0.143
Sand	1/4	1/2	1	3	1/3	2	2	2	4	0.097
Silt	1/5	1/2	1/3	1	1/3	1/3	1/3	1/3	8	0.039
Clay	1/4	1/2	3	3	1	3	3	3	2	0.156
BD	1/4	1/2	1/2	3	1/3	1	2	2	5	0.082
ASWC	1/4	1/2	1/2	3	1/3	1/2	1	2	6	0.07
HC	1/6	1/2	1/2	3	1/3	1/2	1/2	1	7	0.056
Consistency ratio (CR) = 0.064									Sum	1.000
Groundwater quality										
pH	1	1/4	1/4	1/4	1/4				5	0.057
EC	4	1	2	2	2				1	0.345
SAR	4	1/2	1	2	2				2	0.26
Na^+^	4	1/2	1/2	1	1				3	0.169
Cl^–^	4	1/2	1/2	1	1				3	0.169
Consistency ratio (CR) = 0.03									Sum	1.000

See footnote of Table 1.

**Fig 7:**
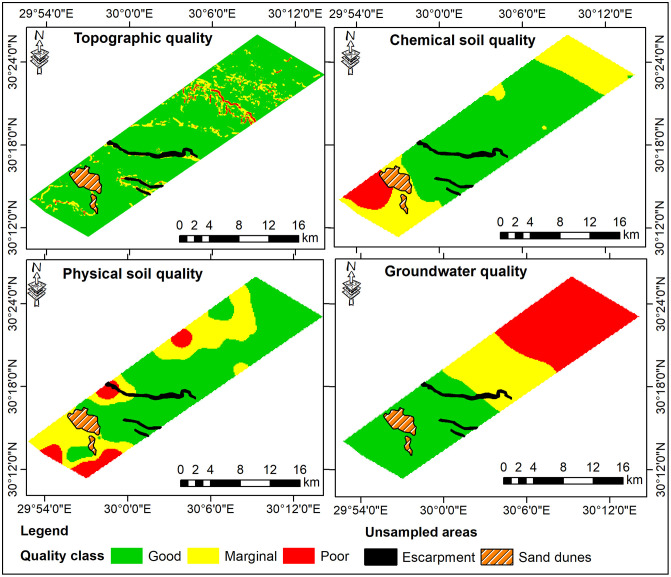
Quality maps of main criteria affecting agricultural potentiality.

As shown in Table 6, 85% of the studied area exhibits good topographic quality, supporting the assertion that slope is not a limiting factor. Moreover, areas with marginal and poor qualities occupied 9 and 2% of the studied region, respectively. Soil with good, marginal, and poor chemical qualities occupied 64, 26, and 26% of the total area, respectively. The quality maps of the four main potentiality criteria are shown in Fig 7. Results reveal that favorable conditions occurred mainly in the middle parts, and the quality level degraded northeast and southwestwards. For soil physical quality, 62, 27, and 7% of the total area were in good, marginal, and poor classes, respectively. The worst conditions were chiefly observed in small patches in the middle and southwestern parts of the studied area. Groundwaters with good, marginal, and poor qualities occurred in 38, 22, and 36% of the studied area, respectively. The proper water quality occurred mainly in the southwestern parts, and fitness for irrigation declined northeastwards.

**Table 6:** Distributions of quality grades for main-criteria in the studied area.

Criterion	Area	Quality grade		
		Good	Marginal	Poor
Topography	km^2^	258.01	27.20	4.80
	%	85.00	8.96	1.58
Chemical soil quality	km^2^	193.11	79.50	17.41
	%	63.62	26.19	5.74
Physical soil quality	km^2^	187.87	80.56	21.58
	%	61.89	26.54	7.11
Groundwater quality	km^2^	114.60	67.13	108.29
	%	37.76	22.12	35.67

**Fig 8:**
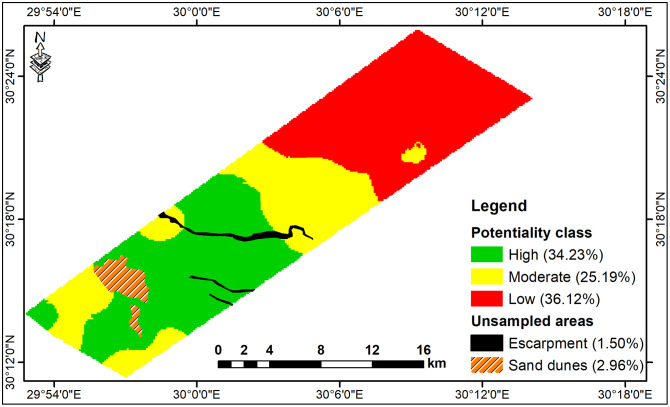
Agricultural potentiality map of the studied area.

The comparison among the selected main criteria indicates varying effects on agricultural potentiality in the studied area. The groundwater quality displayed the highest contribution (50%) followed by soil chemical quality (24%) and soil physical quality (21%), while topographic quality received the lowest influence (5%). The overall potentiality map in the studied region is shown in Fig 8. Result reveals that that 34, 24, and 36% of the total area had high, moderate, and low potentialities for possible agricultural expansion. The highly potential zones occurred in the southwestern parts, while those of low priority were in the northeastern parts.

### 3.4 Model performance assessment

Results of the comparison matrices, shown in Table 5, indicate that values of CR for all implemented models remained below the permissible limit of 10% (0.10). The cross-validation results, presented in Table 7 and S2 File, confirm the reliability of OK models applied to interpolate key soil properties. For all models, values of ME and MSE were near zero, while values of RMSSE were close to unity. In addition, values of RMSE and ASE were comparable; however, they generally exhibited higher values for most of the analyzed properties. This, in turn, advocates for adopting proper sampling strategy to minimize prediction error and maximize prediction accuracy. The prediction of soil pH using the spherical model shows excellent performance (ME = 0.001, RMSSE = 1.016). On the other hand, the prediction of gravel content using the Gaussian model shows higher RMSSE (1.346), indicating potential overestimation of variability.

**Table 7:** Cross-validation of the applied ordinary kriging models.

Property	Model	Prediction error				
		ME	RMSE	MSE	RMSSE	ASE
Soil properties						
pH	Spherical	0.001	0.333	0.002	1.016	0.328
EC, dS m^–1^	Exponential	0.031	8.451	0.002	1.154	7.294
ESP	Exponential	– 0.075	5.590	– 0.013	1.002	5.583
Sand, %	Gaussian	0.009	11.837	0.001	1.017	11.629
Silt, %	Exponential	0.012	12.319	0.005	1.025	11.995
Clay, %	Gaussian	0.074	3.002	0.023	0.959	3.136
OM, g kg^–1^	Exponential	– 0.004	0.909	– 0.004	1.051	0.865
CaCO3, g kg^–1^	Pentaspherical	– 0.086	9.529	– 0.007	1.162	7.963
Gypsum, g kg^–1^	Circular	0.015	0.921	0.014	1.071	0.847
Depth, cm	Spherical	0.004	0.431	0.007	1.084	0.395
Gravel, %	Gaussian	– 0.017	1.778	– 0.046	1.346	1.468
BD, Mg m^–3^	Gaussian	– 0.001	0.103	– 0.011	1.084	0.095
ASWC, %	Pentaspherical	0.000	0.027	0.002	1.009	0.027
HC, cm h^–1^	J-Bessel	0.027	3.908	– 0.064	0.851	4.888
Groundwater properties						
pH	Circular	– 0.001	0.415	–0.002	0.990	0.415
EC, dS m^–1^	Rational Quadratic	0.043	0.920	0.040	0.967	0.960
SAR	Circular	0.011	1.181	0.004	0.999	1.194
Na ^+^ , mmol_C_ L^–1^	Spherical	0.054	4.682	0.009	0.930	5.090
Cl^–^, mmol_C_ L^–1^	Spherical	0.036	8.149	0.003	0.948	8.653
HCO_3_^–^, mmol_C_ L^–1^	Gaussian	0.009	0.366	0.021	1.099	0.329

ME, mean error; RMSE, root mean square error; MSE, mean standardized error; RMSSE, root mean square standardized error; ASE, average standardized error.

## 4 Discussion

### 4.1 Geomorphology and soil formation processes

The geomorphic structure plays a pivotal role in determining potential of agricultural lands. The major landforms provide indications for main soil properties such as slope, depth, texture, structure, and water and nutrient availability [52]. The geomorphological evolution of the studied region, shaped by fluvial deposition, tectonic uplift, and limited aeolian activity, has resulted in landscapes dominated by old alluvial terraces and structural depressions [53]. During fluvial events, the accumulation of the Palaeo-fluvial sediments led to the formation of the old alluvial terraces, which dominate the region [54]. Usually, alluvial landforms are highly preferable for crop production owing to deep, nutrient-rich soils and proper water availability [49]. The tectonic events during the Late Miocene era contributed to the formation of structural landforms; depressions and escarpments [55]. The aeolian activity during the Quaternary era was quite limited [56], and thus dune fields were the least abundant landform. Nevertheless, the presence of dune fields adversely affect land productivity owing to continuous sand deposition, which buries crop and farmlands [57]. The developed landforms, combined with arid climatic conditions, have led to the development of Entisols and Aridisols with limited pedogenic development [38]. The predominance of coarse-textured soils, high gravel content, and low organic matter reflects the prevailing physical weathering processes and sparse vegetation cover typical of desert ecosystems.

### 4.2 Soil physical and chemical constraints

Soils of the studied region exhibited main properties comparable to marginal soils developed in arid desert ecosystems, as proven in earlier case studies [58,59]. These soils are substantially affected by geological formations as well as climate conditions [60]. Owing to limited water availability, the physical weathering of parent rocks serves as the prevailing soil-forming process [61]. Thus, gravel and coarse particles normally dominate the soils, thereby increasing water infiltration and apparent density but decreasing water retention [10]. This, in turn, result in adverse effects on plant growth and development due to low water availability [62]. Our findings affirm those reported by Abuzaid and El-Husseiny [63] and Erian, Nasr [64] who indicated that soils of west Nile Delta region suffer poor physical quality owing to high sand and gravel contents.

The soil chemical quality is heavily affected by interactions of arid conditions with soil parent materials. For instance, the production of hydroxyl ions during the hydrolysis of carbonate salt such as CaCO_3_ and Na_2_CO_3_ can significantly raise the pH levels [65]. The pH rise negatively affect nutrient uptake by plants, reducing crop yield and quality [66]. Additionally, excess sodium carbonate and bicarbonate salts can elevate the level of exchangeable sodium. Consequently, high ESP values cause soil dispersion, reduced permeability, and structure instability [65]. However, the level of soil sodicity in the studied area remains within the permissible level. This is likely due to the presence of high CaCO_3_ content, which releases soluble Ca^2+^ and neutralize the adverse impact of soluble and dissolved Na^+^ in soils [65]. The deep leaching of soluble ions and lime is normally slow in the drylands, causing their accumulation within soil layers [24]. Excessive soil salinity adversely affects crop yield and quality due to osmotic stress, ion toxicity, and nutrient imbalance [67]. Moreover, high lime content induces severe nutritional problems mainly for N, P, Fe, and Zn [68]. The scattered vegetation as well as limit biomass production results in low soil organic carbon content [69]. In arid and semi-arid regions, low organic matter content negatively affects soil fertility and accelerated soil erosion due to structure instability [60]. Our findings are in harmony with those reported in previous case studies [14,64,70], which demonstrated that salinity, sodicity, high lime content, and low organic matter are major constraints of soil quality in west Nile Delta region.

### 4.3 Groundwater quality and salinity risks

Groundwater chemistry further reflects the interaction between geological substrates and anthropic influences, as evidenced in west Nile Delta region [53,63,71]. The abnormal pH value (8.85) is likely due to the presence of toxic ions, which may induce nutritional imbalance. Thus, if applicable, it is necessary to introduce an amendment into the water [46]. Elevated concentrations of bicarbonate and sodium ions, along with high SAR values, suggest ongoing dissolution of carbonate and evaporite minerals, as well as potential contamination from agricultural runoff or domestic source [72]. In drylands, Na^+^ and Cl^–^ are the primary soluble ions in groundwater [73]. These ions are sourced from human activities and the chemical weathering of sodium-feldspar minerals as well as the dissolution of halite (NaCl) [74]. Furthermore, the dissolution of evaporite minerals like gypsum (CaSO_4_.2H_2_O) and anhydrite (CaSO_4_) might contribute to calcium and sulfate ions [75]. Accordingly, few locations in the northeastern parts have experienced elevated levels of salinity in response to high influxes of soluble ions. Our findings are in line with those obtained by Ibraheem, Othman [55] and Hussein, El-Raouf [71], who indicated that groundwater in the west Nile Delta region had a wide range of salinity. Excessive salt in groundwater hinder water uptake by plants due to high osmotic potential. In addition, saline waters with high sodium and chloride ions cause a direct injury from leaf intake and cause nutrient problems [72].

### 4.4 Implications of landscape on agricultural potential

Under irrigated agriculture, the analysis of potential zones in a specific area entails precise information about topography, soil, and irrigation water quality [3,76]. Among a variety of topographic attributes, slope gradient remains the most effective factor. This parameter controls soil-water relationships like irrigation type and rate of drainage [77] and soil erosion potential [51]. Besides, the slope gradient determines cropping patterns and practices [76]. From an agricultural perspective, the region exhibits favorable topographic conditions, with over 94% of the area classified as having high or very high topographic quality. Our finding are in line with those obtained by Abuzaid and El-Husseiny [63], who indicated that the slope gradient in a newly-reclaimed region in west Nile Delta would pose slight limitations for field crop production, i.e., maize, wheat, and broad bean.

Soil quality denotes to the ability of soil to function within its ecosystem to sustain plant and animal productivity, preserve environmental quality, and improve human health [31]. Consequently, the soil ecosystem functions depend mainly on main physicochemical properties, which govern its suitability or fitness for a specific land use [69]. Soil salinity and sodicity, however, remain critical constraints, particularly in marginal zones where EC and ESP values exceed optimal thresholds [72,78,79]. The AHP analysis identified EC, ESP, and soil depth as the most influential factors affecting soil quality. Most of the studied soils had EC values between 2–8 dS m^–1^ (very slightly to slightly saline). Besides, the soils displayed none to slight sodicity hazards (ESP < 15). The soil depth is a key physical property since it governs plant root extension, water logging, and biological activities [69]. Among the studied soil profiles, only five ones had a shallow depth (> 50 cm), while the remaining ones had deep and very deep depth. Hence, approximately two-thirds of the area is characterized by high chemical and physical soil quality, supporting its potential for sustainable agricultural development under appropriate management practices. Soils with poor chemical quality occurred in the outer parts, where soil salinity reached its maximum levels. Soils with poor physical quality (shallow soils) linked with high lime content (strongly and extremely calcareous) in the middle and southwestern parts. This result affirms the findings reported by Abuzaid, Mazrou [10], who indicated that soil depth showed significant negative correlation with lime content in a newly-reclaimed region in Matruh Governorate.

### 4.5 Groundwater quality and agricultural suitability

Groundwater quality is a critical determinant of agricultural productivity in arid regions, as it directly influences soil chemistry and plant health [5]. In this study, EC and SAR were identified as the most influential groundwater parameters, receiving the highest weights in the AHP. Elevated salinity impairs plant water uptake by increasing osmotic pressure, while high SAR compromises soil structure and promotes sodium toxicity [72]. Spatial analysis revealed that groundwater in the southwestern part of the study area, associated with Miocene aquifers, exhibited lower salinity and was thus more suitable for irrigation. Youssef, Ibrahem [53] reported that the Miocene aquifer in the west Nile Delta region usually provides groundwater with low salinity. On the other hand, the worst groundwater condition found in the northeastern parts was related to the Late Pliocene aquifer, which usually provide brackish groundwater [53]. Normally, groundwater with high salinity adversely affects land productivity and crop production on long term [14]. Moreover, it imposes certain irrigation and agricultural practices such as selecting salt-tolerant crops and applying leaching requirements, and application of soil amendments [66].

### 4.6 Overall agricultural potentiality

The AHP results indicated that groundwater quality accounted for 50% of the total agricultural potentiality score, underscoring its dominance over soil (44.5%) and slope (0.5%) in this arid region. This reflects the role of water availability (in terms of quantity and quality) in conjunction with available land resources in achieving SDGs in arid and semi-arid regions [78,80]. Our findings are in line with earlier case studies in west Nile Delta region [14,63], which conformed the superiority of groundwater quality over soil and terrain attributes for predicting crop suitability in west Nile Delta region.

The overall map highlighted that the studied region displayed various agricultural potentials, which has been affirmed by previous cases studies in west Nile Delta region. For instance, Saleh, Elsharkawy [81] implemented several methods for indexing soil quality in a nearby area (Wadi Al-Natrun District). Accordingly, the area had several soil quality grades ranging from grade I (very high) to grade IV (very low) with grade III (moderate) being the dominant class. In the same region, AbdelRahman, Saleh [82] applied the FAO land evaluation framework to assess land suitability for crop production. The study indicated that 21, 41, and 38% of the total area were classified as suitable, marginally suitable, and not suitable. In the same region,

Basically, the degree of land potentiality is directly linked to dominant limitations for agroecosystem functions [29,83]. For instance, high-potential zones were concentrated in the southwest, where both groundwater and soil limitations were minimal. These areas are suitable for a wide range of crops, including moderately salt-sensitive species. The moderate-potential zones were mainly observed in the middle parts and scattered patches across the region, where moderate soil and groundwater limitations occurred. These zones would be suitable for cultivating moderately salt-tolerant crops, including wheat, sorghum, soya bean, cowpea, sunflower, and zucchini [46]. Excessive lime content in these zones imposes integrated soil and water management practices through the proper preparation of soil seedbed using chisel and moldboard plows, addition of chemical amendments, and application of optimum amount of irrigation water. Moreover, foliar fertilization is a potent strategy to manage plant nutrition in calcareous soils [68]. The northeastern zones exhibited greater salinity and sodicity constraints, limiting their suitability to salt-tolerant crops. The recommended crops are olive and other field crops such as canola, barely, sugar beet, and cotton [72]. Moreover, sustainable crop production in areas with marginal qualities entails certain agronomic practices to control salinity hazards. The most effective practices involve computing leaching requirements, land smoothing or grading, application of soil amendments, and balanced fertilization [46].

### 4.7 Model stability and predictive accuracy

The CRs for all AHP comparison matrices were below the threshold of 0.10, confirming the reliability of the weighting scheme as outlined by Saaty [50]. Hence, these models manifested a dependable level of stability and the computed weighting factors for the implied criteria were comparable. Cross-validation of the ordinary kriging (OK) models demonstrated high predictive accuracy, with most RMSSE values approximating 1.0 and minimal mean errors. However, elevated RMSE and ASE values for certain variables suggest the need for refined sampling strategies in future studies. The OK models yielding minimal levels of both ME and MSE provide unbiased estimations at unsampled locations [28]. However, ME > 0 indicates underestimation of data variability, while ME < 0 reflects overestimations [84]. The OK achieving RMSSE values around the optimal value (1.0) reflect high prediction accuracy [85].

## 5 Conclusions

The current work provides a new framework for assessing agricultural potentiality in newly reclaimed arid agroecosystems through co-utilizing AHP, geostatistical, and fuzzy logic techniques under the GIS platform. The applicability of this approach was tested in a typical region in the western Nile Delta fringes of Egypt. The study focused mainly on analyzing the qualities of topography, soil, and groundwater, which serve as the main components of agroecosystems. The consistency ratio for the developed comparison matrices affirmed the feasibility of AHP in allocating reliable weights for main and sub-criteria. Groundwater quality received the greatest importance and accounted for 50%, followed by soil chemical quality (24%) and soil physical quality (21%), while slope gradient contributed to only 0.05%. The EC and ESP were the main drivers for soil chemical quality, while effective depth was the most influential parameter for soil physical quality. The groundwater quality for irrigation was mainly affected by EC and SAR. The cross-validation using prediction errors revealed that the implemented OK geostatistical models demonstrated dependable predictive mapping for soil and groundwater attributes. Soils having high chemical and physical qualities accounted for about 64 and 62% of the total area, respectively. The groundwater quality was mainly affected by water-bearing formations, leading to different quality levels. The overall potentiality map showed that approximately 34, 25, and 36% of the studied region would have high, moderate, and low potentialities, respectively. The proposed methodology would serve as a basic procedure for the comprehensive spatial assessment of agricultural resources using fully automated models. Yet, further validations through adopting an appropriate sampling strategy are advocated to improve the insight into dominant limitations affecting the exploitation of natural resources.

## Supporting information

S1 File Supplementary data. (DOCX)

S2 File Supplementary data. (RAR)
