# Supplementary material for: A GIS-based multi-criteria framework for mapping potential irrigated agricultural zones in newly reclaimed arid agroecosystem
Source: PLoS One. 2026 Jun 26;21(6):e0351546. doi: 10.1371/journal.pone.0351546 (PMC13308839; doi:10.1371/journal.pone.0351546)
Supplement: S1 File — (DOCX) [file pone.0351546.s001.docx]

**Supplementary materials**

**A GIS-based multi-criteria framework for mapping potential irrigated agricultural zones in newly reclaimed arid agroecosystem**

Ahmed S. Abuzaid^1^, Hassan H. Abbas^1^, Yousif K. El Ghonamy^2^, Mostafa A. Mostafa^1^, Nazih Y. Rebouh^3^, Mohamed S. Shokr^4^

^1^ Soils and Water Department, Faculty of Agriculture, Benha University, Benha 13518, Egypt

^2^ Soils, Water, and Environment Research Institute (SWERI), Agricultural Research Center (ARC), Giza 12619, Egypt

^3^ Department of Environmental Management, Institute of Environmental Engineering, RUDN University, 6 Miklukho-Maklaya Street, 117198 Moscow, Russia

^4^ Soil and Water Department, Faculty of Agriculture, Tanta University, Tanta 31527, Egypt

***** Correspondence: mohamed_shokr@agr.tanta.edu.eg

**Table S1: FAO 29 guidelines for interpretations of water quality for irrigation**

| **Potential irrigation problem** | | | | **Unit** | **Degree of restriction on use** | | |
| --- | --- | --- | --- | --- | --- | --- | --- |
| **Salinity** (affects crop water availability) | | | | | **None** | **Slight to moderate** | **Severe** |
|  | EC_w_ |  |  | dS m^–1^ | 0.7 | 0.7 – 0.3 | < 3.0 |
|  | TDS |  |  | mg L^–1^ | > 450 | 450 – 2000 | < 2000 |
| **Infiltration** (affects infiltration rate of water into the soil. Evaluate using EC_w_ and SAR together) | | | |  |  |  |  |
| SAR | = 0 – 0.3 | EC_w_ | = |  | < 0.7 | 0.7 – 0.2 | > 0.2 |
|  | = 3 – 6 |  | = |  | < 1.2 | 1.2 – 0.3 | > 0.3 |
|  | = 6 – 12 |  | = |  | < 1.9 | 1.9 – 0.5 | > 0.5 |
|  | = 12 – 20 |  | = |  | < 2.9 | 2.9 – 1.3 | > 0.1.3 |
|  | = 20 – 40 |  | = |  | < 5.0 | 5.0 – 2.9 | > 2.9 |
| **Specific ion toxicity** (affects sensitive crops) | | | | |  |  |  |
|  | **Sodium (Na^+^)** | | |  |  |  |  |
|  | Surface irrigation | | | SAR | > 3 | 3 – 9 | < 9 |
|  | Sprinkler irrigation | | | mmol_c_ L^–1^ | > 3 | < 3 |  |
|  | **Sodium (Cl^–^)** | | |  |  |  |  |
|  | Surface irrigation | | | SAR | > 4 | 4 – 10 | < 10 |
|  | Sprinkler irrigation | | | mmol_c_ L^–1^ | > 3 | < 3 |  |
| **Miscellaneous effects** (affects susceptible crops) | | | | |  |  |  |
|  | **Bicarbonate (HCO_3_^–^)** | | |  |  |  |  |
|  | (Overhead sprinkling only) | | | mmol_c_ L^–1^ | > 1.5 | 1.5 – 8.5 | < 8.5 |
|  | **pH** | | |  | Normal range 6.5 – 8.4 | | |

Adapted from Ayers and Westcot (1)

| Semivariogram of pH | Semivariogram of EC |
| --- | --- |
| 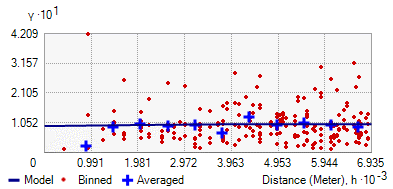 | 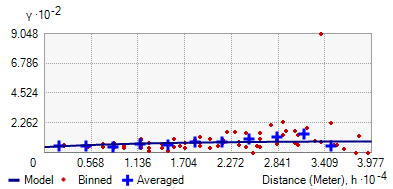 |
| Semivariogram of ESP | Semivariogram of sand content |
| 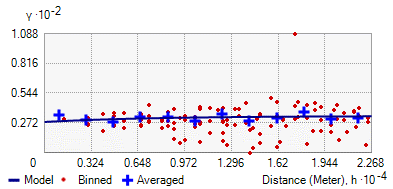 | 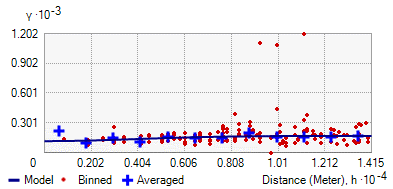 |
| Semivariogram of silt content | Semivariogram of clay content |
| 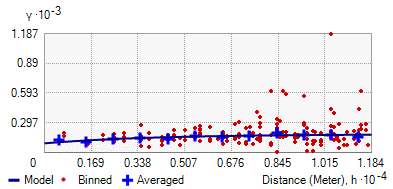 | 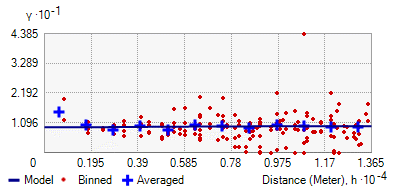 |
| Semivariogram of OM content | Semivariogram of CaCO_3_ content |
| 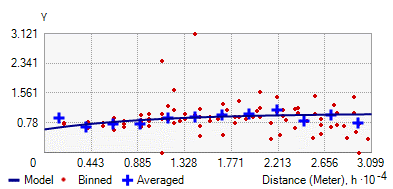 | 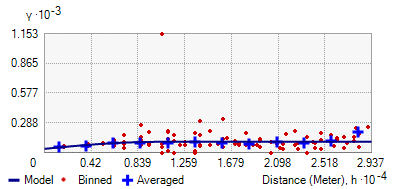 |

Fig. S1. Experimental semivariograms and their best-fitted models for soil attributes

| Semivariogram of gypsum content | Semivariogram of depth |
| --- | --- |
| 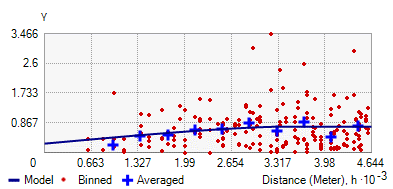 | 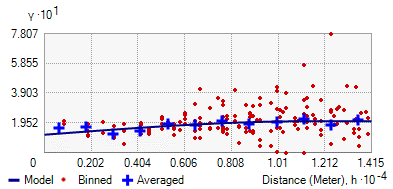 |
| Semivariogram of coarse fragments | Semivariogram of bulk density |
| 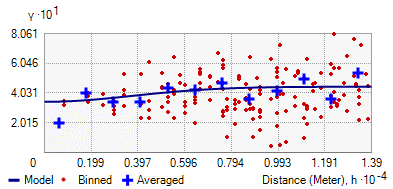 | 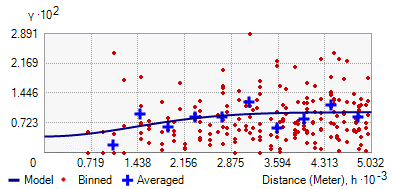 |
| Semivariogram of available water content | Semivariogram of hydraulic conductivity |
| 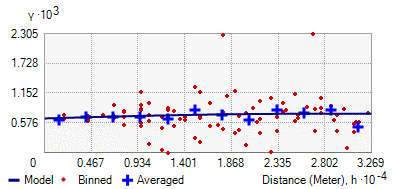 | 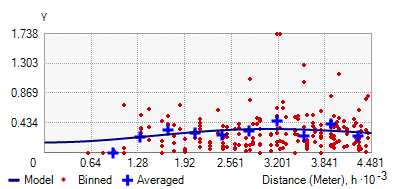 |

Fig. S1. Continuous

| Cross-validation for pH | Cross-validation for EC |
| --- | --- |
| 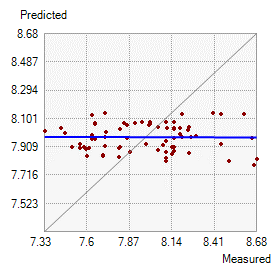 | 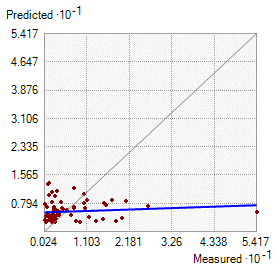 |
| Cross-validation for ESP | Cross-validation for sand content |
| 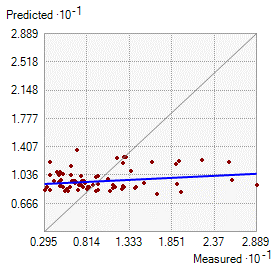 | 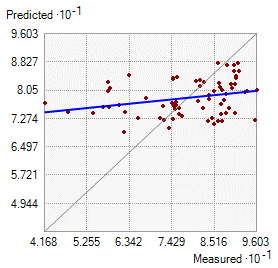 |
| Cross-validation for silt content | Cross-validation for clay content |
| 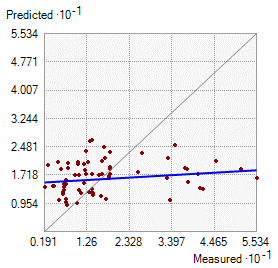 | 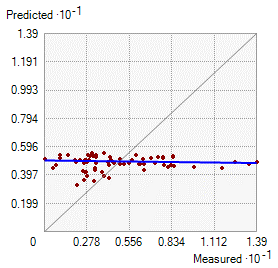 |

Fig. S2. Cross-validation test of the applied ordinary kriging models for soil attributes

| Cross-validation for OM content | Cross-validation for CaCO_3_ content |
| --- | --- |
| 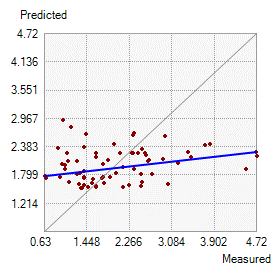 | 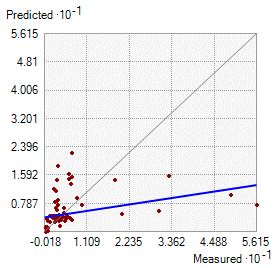 |
| Cross-validation for gypsum cotent | Cross-validation for depth |
| 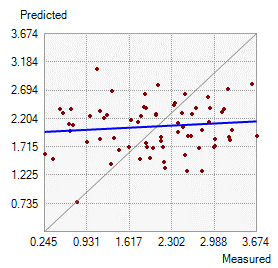 | 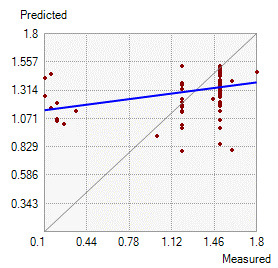 |
| Cross-validation for corase fragement content | Cross-validation for bulk density |
| 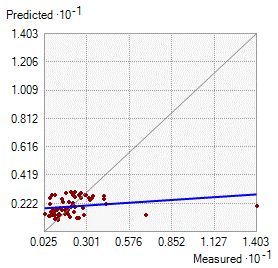 | 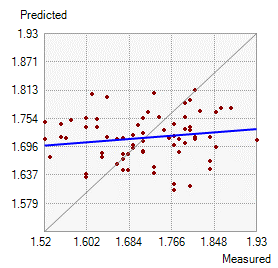 |

Fig. S2. Continuous

| Cross-validation for available water content | Cross-validation for hydraulic conductivity |
| --- | --- |
| 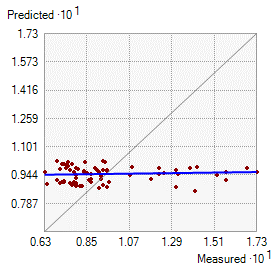 | 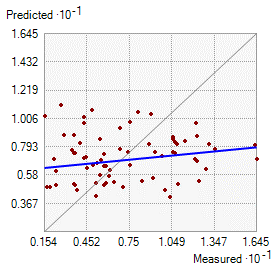 |

Fig. S2. Continuous

| Semivariogram of pH | Semivariogram of EC |
| --- | --- |
| 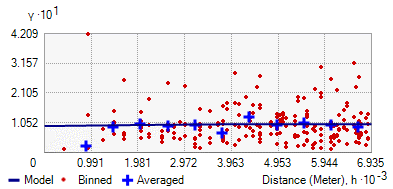 | 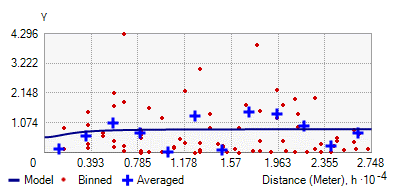 |
| Semivariogram of SAR | Semivariogram of Na^+^ |
| 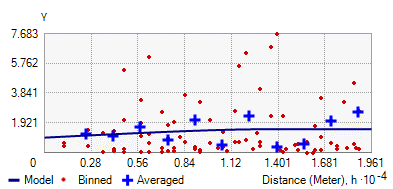 | 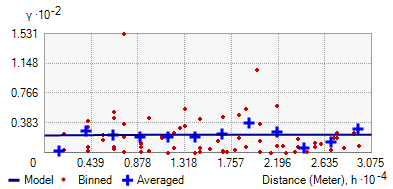 |
| Semivariogram of Cl^–^ | Semivariogram of HCO_3_^–^ |
| 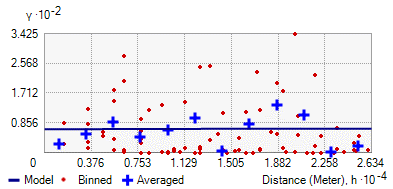 | 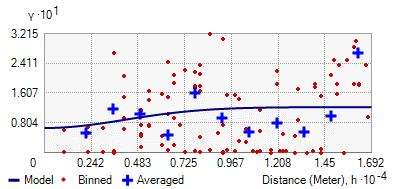 |

Fig. S3. Experimental semivariograms and their best-fitted models for groundwater attributes

| Cross-validation for pH | Cross-validation for EC |
| --- | --- |
| 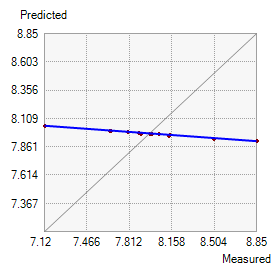 | 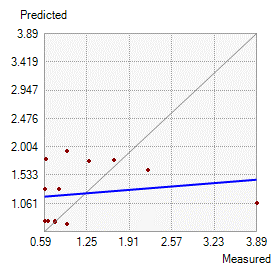 |
| Cross-validation for SAR | Cross-validation for Na^+^ |
| 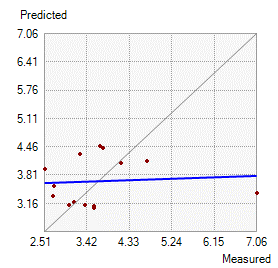 | 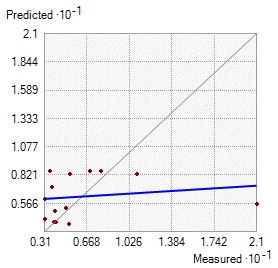 |
| Cross-validation for Cl^–^ | Cross-validation for HCO_3_^–^ |
| 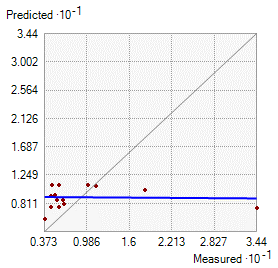 | 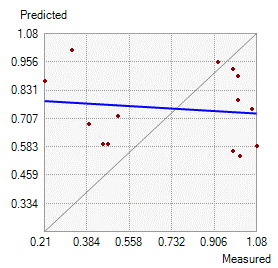 |

Fig. S4. Cross-validation test of the applied ordinary kriging models for groundwater attributes

References

1. Ayers RS, Westcot DW. Water quality for agriculture. FAO Irrigation and Drainage Paper 29. Rome, Italy: Food and Agriculture Organization of the United Nations (FAO); 1994.
